# New IOL formula using anterior segment three-dimensional optical coherence tomography

**DOI:** 10.1371/journal.pone.0236137

**Published:** 2020-07-20

**Authors:** Young-Sik Yoo, Woong-Joo Whang, Hyun-Seung Kim, Choun-Ki Joo, Geunyoung Yoon

**Affiliations:** 1 Department of Ophthalmology, Uijeongbu St. Mary’s Hospital, College of Medicine, The Catholic University of Korea, Gyeonggi-do, South Korea; 2 Department of Ophthalmology, Yeouido St. Mary’s Hospital, College of Medicine, The Catholic University of Korea, Seoul, South Korea; 3 Department of Ophthalmology, Seoul St. Mary’s Hospital, College of Medicine, The Catholic University of Korea, Seoul, South Korea; 4 CK St. Mary Eye Center, Seoul, South Korea; 5 Flaum Eye Institute, The Institute of Optics, Center for Visual Science, University of Rochester, Rochester, New York, United State of America; Universidad de Monterrey Division de Ciencias de la Salud, MEXICO

## Abstract

This retrospective study was aimed to compare prediction errors from various combinations of biometric data generated using optical coherence tomography (OCT) and develop a new intraocular lens (IOL) formula using biometric data. 145 eyes from 145 patients who underwent femtosecond laser-assisted cataract surgery (FLACS) were enrolled to the present study and they were divided into a training set (n = 92) and a test set (n = 53). Preoperative axial length (AL) and corneal radius were measured using partial coherence interferometry. The anterior chamber depth (ACD), lens meridian parameter (LMP), lens thickness (LT), thickness of anterior and posterior parts of the crystalline lens (aLT and pLT), and anterior segment length were measured by OCT. From a training set, we developed eight regression equations and analyzed the predictive accuracy. The regression equation using AL, LMP, and pLT (-1.143 + 0.148*AL + 0.428*LMP + 0.254*pLT) showed the strongest correlation with effective lens position (ELP) and smallest standard deviation of ELP prediction error. IOL formula generated using AL, LMP, and pLT yielded the highest predictive accuracy. In a test set, the new IOL formula also produced narrowest range of prediction error, smallest median absolute error, and highest percentages within ±0.25, ±0.50 than existing IOL formulas. The IOL formula considering AL, LMP and pLT will help to improve predictive accuracy in FLACS.

## Introduction

Modern cataract surgery is considered to be a combined rehabilitative and refractive procedure, and is known as refractive cataract surgery. Patient expectations for optimal refractive outcomes have increased along with improvements in surgical techniques and intraocular lens (IOL) technology. The postoperative refractive outcome is the most important factor for patient satisfaction [1].

An effective lens position (ELP) is not a physical distance with thin lens formulas and cannot be measured, as it is the distance between the cornea and the secondary principal plane of the IOL. The prediction of an ELP is the most important process in IOL power calculation [2]. In 1988, Holladay proposed a direct relationship between the steepness of the cornea and position of the IOL. The distance from the iris plane to the IOL is known as the surgeon factor (SF) and is specific to each lens. The Haigis formula, one of the fourth-generation IOL formulas, used preoperative ACD measurements to predict ELP instead of corneal steepness.

Optical coherence tomography (OCT) is a non-invasive, high-resolution imaging technology that provides in vivo cross-sectional images of ocular structures [3, 4]. The use of OCT is progressively increasing because it provides accurate measurements and is fast, safe and comfortable for both patients and operators. Recent studies have demonstrated that the application of anterior segment-optical coherence tomography (AS-OCT) is useful in preoperative planning for cataract surgery [5–8]. Detailed knowledge about the shape and thickness of the crystalline lens is critical when femtosecond laser-assisted cataract surgery (FLACS) is considered. Indeed, some advanced FLACS platforms include OCT-based systems to identify and measure lens structures. Notably, the Catalys Precision Laser System includes 3-dimensional OCT (3D-OCT) and measures axial and sagittal sectional scanned images.

This study aimed to compare the prediction errors from various combinations of biometric data provided by Catalys 3D-OCT and develop a new IOL formula using these biometric data. We also compared the predictive accuracy of a new IOL formula with existing IOL formulas in an external test sample.

## Materials and methods

### Subjects

This retrospective study included 145 eyes from 145 patients who underwent femtosecond laser-assisted cataract surgery (FLACS) from Jan 2016 to May 2017. Exclusion criteria were previous ocular surgery, corneal diseases, pseudoexfoliation, zonular weakness, corneal astigmatism greater than 1.00 diopters, glaucoma, macular disease, and amblyopia. Eyes with best-corrected distant vision less than 20/40 in the postoperative state were also excluded. Total 145 eyes were divided into a training set (92 eyes with FLACS from Jan 2016 to Sep 2016) and a test set (53 eyes with FLACS from Nov 2016 to May 2017). The study protocol adhered to the tenets of the Declaration of Helsinki for the use of human participants in biomedical research. The Institutional Review Board (IRB #KC13DISI0534) for Human Studies, Seoul St. Mary Hospital approved this study.

#### Patient examinations

**Fig 1:**
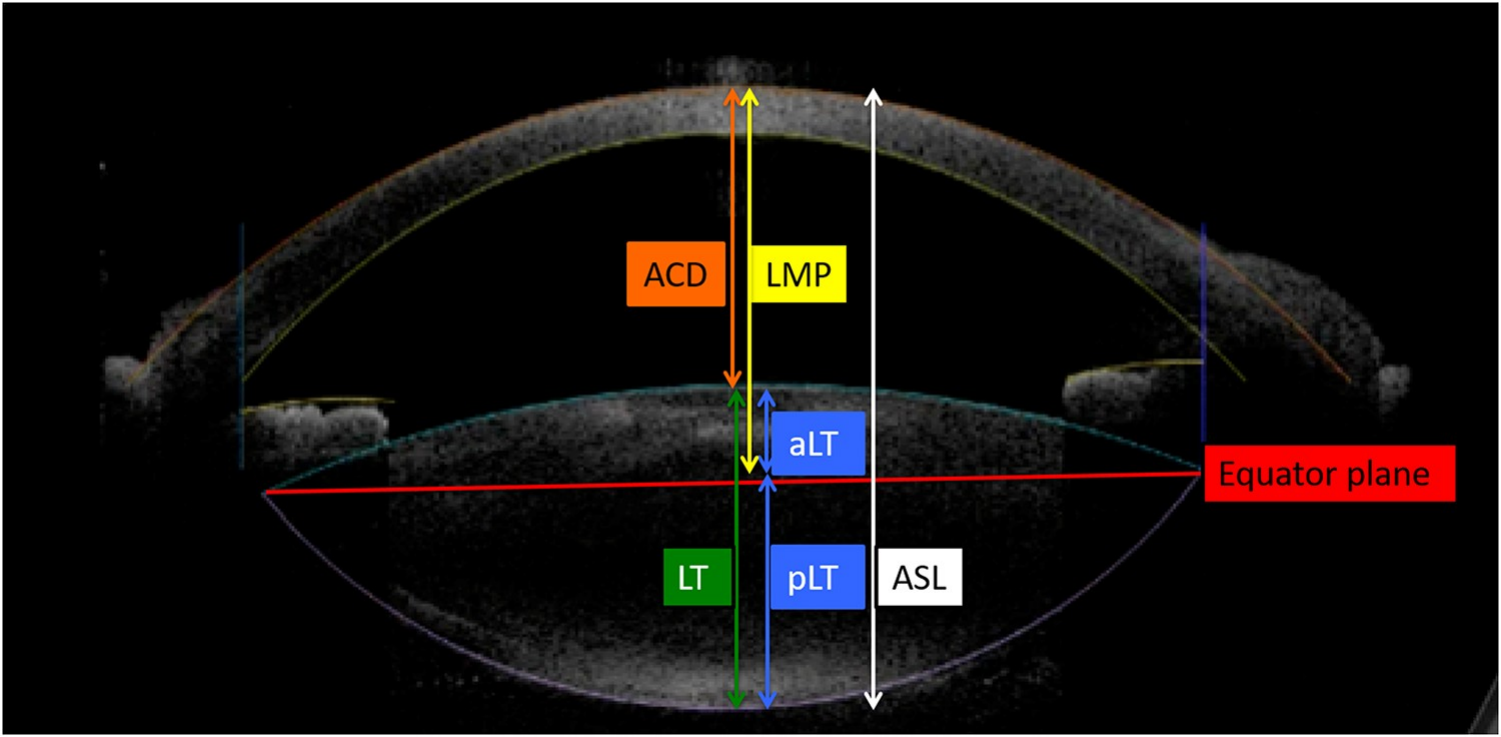
Biometric measurements provided by 3-dimensional optical coherence tomography (3-D OCT). The 3-D OCT images show the scanned capsule, which is an imaginary line of the crystalline lens visualized from the anterior and posterior capsule, provided by the built-in algorithm of the laser system. The equator plane is a straight line that connects both ends of the imaginary lines. Anterior chamber depth (ACD) is determined by calculating the distance along the visual axis between the corneal epithelium and anterior lens surface by OCT. The lens meridian parameter (LMP) is the vertical distance from the corneal apex to the equator plane of the crystalline lens. The anterior segment length (ASL) was calculated by adding the lens thickness (LT) to the ACD. The crystalline lens was also analyzed by dividing it into an anterior part (aLT) and posterior part (pLT), bordered by the equator plane.

Preoperative measurements were performed with the IOLMaster 5.4 (Carl-Zeiss Meditec, Germany). The IOLMaster uses partial coherence interferometry to measure the axial length (AL). Corneal power is measured by automated keratometry, which should be performed first because the system requires the input of corneal radii to calculate the anterior chamber depth (ACD). The ACD is determined by calculating the distance along the visual axis between the corneal epithelium and anterior lens surface using lateral slit illumination. Fig 1 shows biometric measurements provided using 3D-OCT with a Catalys Precision Laser System. It measures the anterior chamber depth (ACD), lens thickness (LT), and lens meridian parameter (LMP). The LMP is defined as the length from the anterior surface of the central cornea to the crystalline lens equatorial plane. The equatorial lens plane was determined by two meridians of the lens equator, which was estimated based on an imaginary line connecting the anterior and posterior capsule surface imaged with 3-D OCT in a femtosecond laser machine for cataract surgery. We also evaluated anterior segment length (ASL), the anterior part of crystalline lens (aLT), and the posterior part of crystalline lens (pLT). The aLT and pLT were classified based on the boundary of the lens equator plane. In the calculation process of ASL, ACD measured by 3D-OCT was used.

### Surgical technique

All procedures were performed by the two surgeons (WJW and CKJ). After sufficient pupillary dilation was confirmed, femtosecond laser pretreatments were performed with the Catalys Precision Laser System (Abbott Medical Optics, Abbott Laboratories Inc., Abbott Park, IL). The disposable vacuum interface (LIQUID OPTICS, Abbott Medical Optics, Abbott Laboratories Inc., Abbott Park, IL) was positioned and fixed to the globe using a suction ring and the laser aperture was engaged with the vacuum interface-globe complex. After completing the entire laser emission procedure, the vacuum interface was removed. The parameters for femtosecond laser-assisted pretreatment are described in Table 1. At the initiation of cataract surgery, the incised circular capsule (5.2 mm in diameter, scanned capsule type) was removed using micro-forceps. All surgeries were performed using an Ozil torsional hand piece with the Infiniti Vision System (Alcon). Following phacoemulsification, one type of intraocular lens (ZCB00, Johnson & Johnson Vision Care, Inc.) was inserted into the capsular bag. No intraoperative complications occurred.

**Table 1:** Parameters for femtosecond laser-assisted pretreatment.

Procedures	Parameters	Value
Capsulotomy	Size (mm)	5.2
	Pulse energy (μJ)	4
	Spot spacing (horizontal/vertical, μm)	5 / 10
Lens fragmentation	Pattern	Quadrant
	with softening	
	Pulse energy (anterior/posterior, μJ)	8 / 10
	Spot spacing (horizontal/vertical, μm)	10 / 40
	Segmentation repetitions (n)	4
Primary incision	Segmentation spacing (μm)	500 / 500
	Pulse energy (μJ)	6
	Spot spacing (horizontal/vertical, μm)	4 / 8
Sideport incision	Width / length (mm)	2.3 / 1.1
	Pulse energy (μJ)	6
	Spot spacing (horizontal/vertical, μm)	3 / 5

#### New equations for ELP prediction

New equations for ELP prediction were developed in a training set (n = 92). Refractive outcomes were measured three months postoperatively with manual refraction and ELP was back-calculated using the following thin-lens formula [9, 10]:

$$\text{IOL}\;\text{power}=\frac{1336}{\text{AL}-\text{ELP}}-\frac{1336}{\;\frac{1336}{Z}–\text{ELP}}$$

$$Z=\frac{\left(\text{nc}-1\right)1000}{\text{r}}+\frac{1000}{\frac{1000}{PostRx.}-\text{VD}}$$

where AL is the preoperative axial length, ELP is the effective lens position, nc is the fictitious corneal refractive index (1.3315), r is the preoperative corneal radius, PostRx. is the postoperative refraction, and VD is the vertex distance.

We obtained AL and ACD from PCI. ACD, LMP, LT, aLT, pLT and ASL were measured by 3D-OCT. From these variables, we developed eight combinations including existing Haigis formula: combination of AL and ACD. These combinations are listed in Table 2. New regression formulas for predicting ELP were made for each combination.

**Table 2:** The combinations of biometric measurement for effective lens position (ELP) prediction and intraocular lens power calculation.

Preoperative variables for ELP prediction
Axial length, ACD (by PCI)
Axial length, ACD (by 3D-OCT)
Axial length, LMP
Axial length, ACD, aLT
Axial length, ACD, LT
Axial length, LMP, pLT
Axial length, ASL
Axial length, ACD, aLT, pLT

ACD = anterior chamber depth; PCI = partial coherence interferometry; 3D-OCT = 3-dimensional optical coherence tomography; LMP = lens meridian parameter; aLT = anterior part of lens thickness; LT = lens thickness; pLT = posterior part of lens thickness; ASL = anterior segment length.

### Main outcome measures

The ELP prediction error was defined as the value obtained by subtracting the ELP predicted by each combination from the back-calculated ELP using the thin-lens formula [9, 10]. The prediction error in refraction was the actual postoperative spherical equivalent (SE) minus the predicted SE, while the mean error (ME) was the mean value of the prediction error. The mean absolute error (MAE) and median absolute error (MedAE) were the mean value and median value of the absolute value of the ME. We also calculated the percentage of eyes with a ME of ±0.25, ±0.50, and ±1.00 diopters or less. Additionally, we analyzed associations between preoperative lens thickness and prediction error for each combination. We selected six kinds of IOL formulas and the predictive accuracy of them were compared with the new IOL formula in a test set. The IOL constant of each formula were as follows;

The Barret-Universal II: lens factor = 2.09; the Haigis: a0 = -1.302 / a1 = 0.210 / a2 = 0.251; the Hill-RBF: A = 119.34; the Hoffer Q: pACD = 5.80: the SRK/T: A = 119.3; the T2: A = 119.3. The predictive accuracy was also investigated in a test set.

### Statistical analysis

Multiple linear regression tests were used to develop eight kinds of ELP prediction equations and determine improvements in the total R^2^ value. Pearson’s correlation tests were performed to determine the strength of association between preoperative lens thickness and the mean error from each combination. Student t-test was used to determine the significance of differences between the training set and the test set. Friedman test was also performed for the comparison between 8 combinations in the training set and the comparison between the new IOL formula and the existing formulas, for the MAE and MedAE. Statistical analyses were performed using SPSS statistical software (version 23.0, SPSS, Inc., Chicago, IL, USA) and statistical significance was defined as *P* < 0.05.

## Results

### New IOL calculation formula from a training set

Mean patient age was 67.1 ± 9.7 (SD) years (range: 48 to 90 years); 60 (65.2%) patients were women and 32 (34.8%) were men. Table 3 shows biometric measurements obtained using PCI and 3D-OCT.

**Table 3:** Clinical characteristics of patients with femtosencond laser-assisted cataract surgery in training set (n = 92).

		Mean	Min.	Max.
3D-OCT	ACD (mm)	3.30 ± 0.40	2.1	4.3
	LMP (mm)	4.82 ± 0.32	4.1	5.5
	LT (mm)	4.60 ± 0.42	3.4	5.6
	ASL (mm)	7.91 ± 0.31	7.1	8.7
	aLT (mm)	1.52 ± 0.29	0.8	2.1
	pLT (mm)	3.08 ± 0.31	2.5	4.1
PCI	ACD (mm)	3.15 ± 0.37	2.2	3.9
	AL (mm)	23.87 ± 1.19	21.41	28.55
	CR (mm)	7.66 ± 0.27	7.03	8.23
IOL power (diopter)		20.90 ± 2.57	12.0	26.0

3D-OCT = 3-dimensional optical coherence tomography; ACD = anterior chamber depth; LMP = lens meridian parameter; LT = lens thickness; ASL = anterior segment length; aLT = anterior part of lens thickness; pLT = posterior part of lens thickness; PCI = partial coherence interferometry; AL = axial length; CR = corneal radius; IOL = intraocular lens.

Table 4 shows regression formulas for the ELP prediction and ELP prediction error of each combination. Instead of the ACD that is used in the existing Haigis formula, combinations involving LMP showed a stronger association in multiple linear regression analysis (combination of AL and ACD: adjusted R^2^ value = 0.36 versus combination of AL and LMP: adjusted R^2^ value = 0.40). Notably, the combinations of AL, LMP, and pLT, and of AL, ACD, aLT and pLT both showed the strongest correlation and smallest standard deviation of ELP prediction error (R^2^ = 0.43, ELP error = 0.00 ± 0.28, respectively).

**Table 4:** Regression formulas for effective lens position (ELP) prediction and ELP prediction error from each combination from training set (n = 92).

Preoperative variables for ELP prediction	Regression formula for ELP prediction	Adjusted R square value	*P* value	ELP prediction error
AL, ACD (by PCI)	1.236 + 0.141*AL + 0.205*ACD	0.36	<0.001	0.00 ± 0.30
AL, ACD (by 3D-OCT)	1.123 + 0.152*AL + 0.149*ACD	0.36	<0.001	0.00 ± 0.30
AL, LMP	0.248 + 0.148*AL + 0.304*LMP	0.40	<0.001	0.00 ± 0.29
AL, ACD, aLT	0.122 + 0.153*AL + 0.292*ACD + 0.334*aLT	0.40	<0.001	0.00 ± 0.29
AL, ACD, LT	-1.090 + 0.151*AL + 0.382*ACD + 0.317*LT	0.42	<0.001	0.00 ± 0.28
AL, LMP, pLT	-1.143 + 0.148*AL + 0.428*LMP + 0.254*pLT	0.43	<0.001	0.00 ± 0.28
AL, ASL	-1.317 + 0.163*AL + 0.338*ASL	0.41	<0.001	0.00 ± 0.28
AL, ACD, aLT, pLT	-1.227 + 0.152*AL + 0.418*ACD + 0.449*aLT + 0.254*pLT	0.43	<0.001	0.00 ± 0.28

AL = axial length; ACD = anterior chamber depth; PCI = partial coherence interferometry; 3D-OCT = 3-dimensional optical coherence tomography; LMP = lens meridian parameter; aLT = anterior part of lens thickness; LT = lens thickness; pLT = posterior part of lens thickness; ASL = anterior segment length.

Predictive accuracy derived from each combination is listed in Table 5. The combination of AL, LMP, and pLT yielded the smallest standard deviation of ME, narrowest range of ME, smallest maximum error, smallest MAE, smallest MedAE, and highest percentages within ±0.25, ±0.50, and ±1.00 diopters.

**Table 5:** Predictive accuracy derived from each combination in a training set (n = 92).

Preoperative variables for ELP prediction		ME	Range of ME	MAE	MedAE	The percentages of ME within		
						±0.25	±0.50	±1.00
Haigis	AL, ACD (by PCI)	0.00 ± 0.40	-0.80 ~ 1.12	0.33 ± 0.24	0.29	43.5	81.5	97.8
	AL, ACD (by 3D-OCT)	0.00 ± 0.40	-0.81 ~ 1.13	0.32 ± 0.24	0.29	43.5	80.4	97.8
AL, LMP		0.00 ± 0.39	-0.78 ~ 0.99	0.31 ± 0.22	0.28	41.3	81.5	100.0
AL, ACD, aLT		0.00 ± 0.39	-0.80 ~ 0.99	0.32 ± 0.22	0.28	42.4	80.4	100.0
AL, ACD, LT		0.00 ± 0.38	-0.81 ~ 0.94	0.31 ± 0.22	0.25	50.0	82.6	100.0
AL, LMP, pLT		0.00 ± 0.37	-0.77 ~ 0.89	0.30 ± 0.21	0.25	50.0	82.6	100.0
AL, ASL		0.00 ± 0.38	-0.88 ~ 0.95	0.31 ± 0.22	0.25	50.0	79.3	100.0
AL, ACD, aLT, pLT		0.00 ± 0.39	-0.88 ~ 0.95	0.31 ± 0.22	0.26	48.9	79.3	100.0
*P value		<0.001		0.95				

ACD = anterior chamber depth; LMP = lens meridian parameter; ASL = anterior segment length; aLT = anterior part of lens thickness; pLT = posterior part of lens thickness; AL = axial length.

*P value by Friedman test.

**Fig 2:**
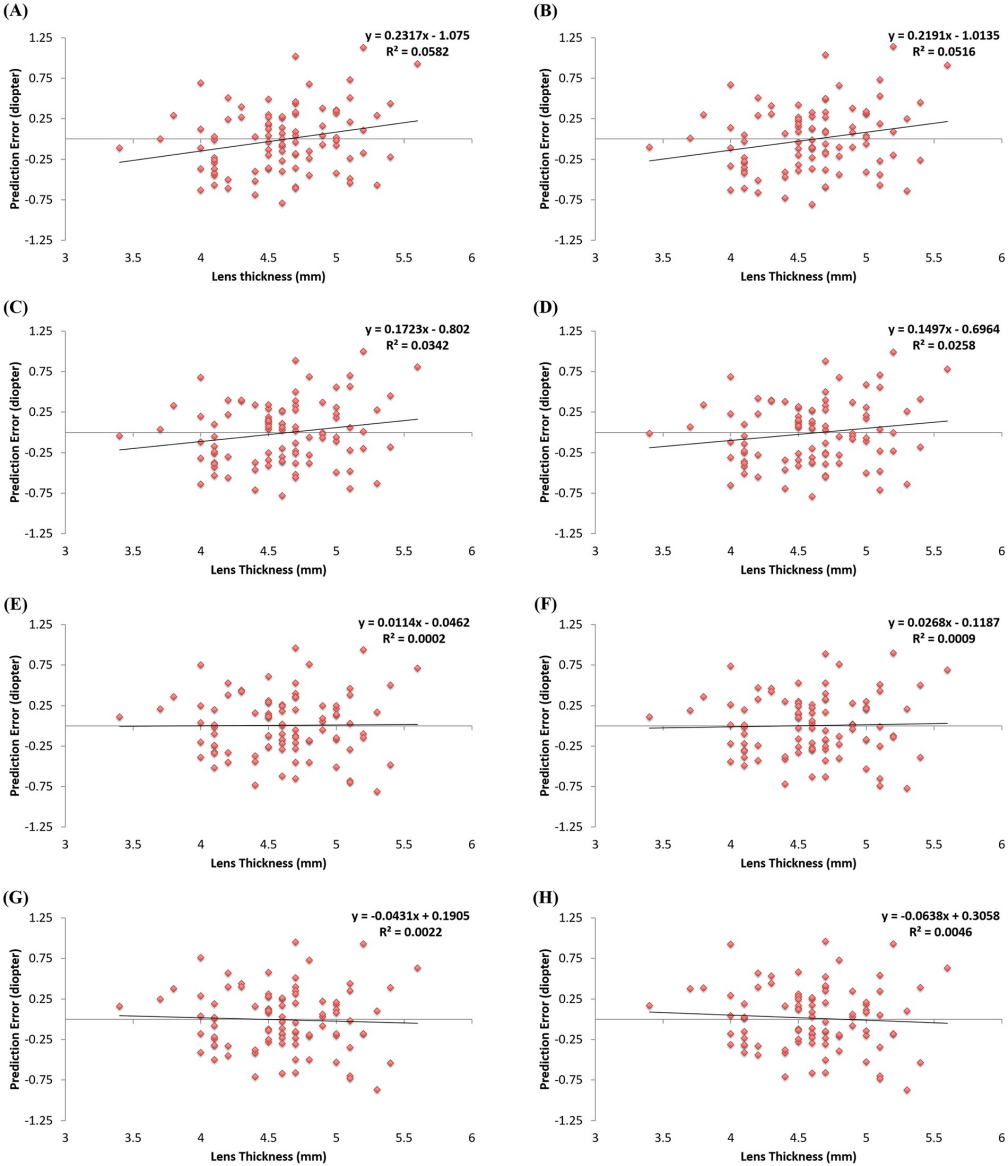
Correlation of preoperative lens thickness with prediction error derived from each intraocular lens formula in training set (n = 92). AL and ACD by PCI (A), AL and ACD by AS 3-D OCT (B), AL and LMP (C), AL, ACD, and aLT (D), AL, ACD, and LT (E), AL, LMP, and pLT (F), AL and ASL (G), AL, ACD, aLT, and pLT (H). AL = axial length; ACD = anterior chamber depth; PCI = partial coherence interferometry; AS 3-D OCT = anterior segment 3-dimensional optical coherence tomography; LMP = lens meridian parameter; aLT = anterior part of lens thickness; LT = lens thickness; pLT = posterior part of lens thickness; ASL = anterior segment length.

Fig 2 shows the correlation between preoperative LT and prediction error derived from each combination. The combinations of AL and ACD show significant negative correlations with preoperative LT (r = 0.23, P = 0.021 for AL and ACD by PCI; r = 0.22, P = 0.030 for AL and ACD by 3D-OCT). The Haigis formula, results in a more hyperopic result when the preoperative cataractous lens becomes thicker. On the other hand, prediction errors in six other combinations using LMP, LT, aLT, pLT, and ASL did not show a significant correlation with preoperative LT.

### Comparison with existing IOL calculation formulas in a test set

Mean patient age was 69.2 ± 9.7 (SD) years (range: 49 to 89 years); 33 (62.3%) patients were women and 20 (37.7%) were men. There was no significant difference in mean age of the study population. (*P* = 0.24) Demographic data in a test set are listed in Table 6. There was no significant difference between a training set and a test set. As the combination of AL, LMP, and pLT produced the highest predictive accuracy in the training set (narrowest standard deviation of ME, narrowest range of ME, smallest maximum error, smallest MAE, smallest MedAE, and highest percentages within ±0.25, ±0.50, and ±1.00 diopters), we chose the above combination for new IOL formula.

ELP=−1.143+0.148*AL+0.428*LMP+0.254*pLT

**Table 6:** Clinical characteristics of patients with femtosencond laser-assisted cataract surgery in the test set (n = 53).

		Mean	Min.	Max.	*P value
3D-OCT	ACD (mm)	3.32 ± 0.37	2.5	4.1	0.87
	LMP (mm)	4.86 ± 0.42	4.1	5.8	0.55
	LT (mm)	4.68 ± 0.56	3.6	5.7	0.38
	ASL (mm)	7.99 ± 0.60	6.7	9.2	0.32
	aLT (mm)	1.55 ± 0.25	0.7	2.2	0.57
	pLT (mm)	3.14 ± 0.60	2.1	4.1	0.56
PCI	ACD (mm)	3.18 ± 0.39	2.0	4.1	0.67
	AL (mm)	23.83 ± 0.99	22.05	26.99	0.85
	CR (mm)	7.60 ± 0.19	7.18	7.94	0.16
IOL power (diopter)		20.47 ± 2.41	12.5	26.0	0.37

3D-OCT = 3-dimensional optical coherence tomography; ACD = anterior chamber depth; LMP = lens meridian parameter; LT = lens thickness; ASL = anterior segment length; aLT = anterior part of lens thickness; pLT = posterior part of lens thickness; PCI = partial coherence interferometry; AL = axial length; CR = corneal radius; IOL = intraocular lens.

* *P* value by Student t-test (Comparison with the training set).

Table 7 shows comparison of the predictive accuracy between the new IOL formula and existing IOL formulas. New IOL formula produced the narrowest range of ME, smallest MAE, smallest MedAE, and highest percentages within ±0.25 and ±0.50 diopters.

**Table 7:** Predictive accuracy derived from new intraocular lens (IOL) calculation formula compared with the accuracy of the Barrett-Universal II, Haigis, Hill-RBF, Hoffer Q, SRK/T, and T2 formulas in test set (n = 53).

Formula	ME	Range of ME	MAE	MedAE	The percentages of ME within		
					±0.25	±0.50	±1.00
New IOL formula using 3D-OCT	-0.01 ± 0.35	-0.70 ~ 0.69	0.29 ± 0.20	0.26	45.3	83.0	100.0
Barrett-Universal II	-0.08 ± 0.36	-0.80 ~ 0.74	0.31 ± 0.20	0.28	41.5	81.1	100.0
Haigis	-0.13 ± 0.40	-1.06 ~ 0.77	0.35 ± 0.23	0.32	35.8	73.6	98.1
Hill-RBF	-0.16 ± 0.37	-0.95 ~ 0.69	0.35 ± 0.21	0.31	39.6	75.5	100.0
Hoffer Q	-0.10 ± 0.41	-1.13 ~ 0.83	0.34 ± 0.25	0.34	43.4	73.6	98.1
SRK/T	-0.26 ± 0.40	-1.07 ~ 0.51	0.40 ± 0.26	0.42	35.8	67.9	98.1
T2	-0.18 ± 0.38	-1.01 ~ 0.66	0.36 ± 0.23	0.34	35.8	75.5	98.1

ME = mean error; MAE = mean absolute error; MedAE = median absolute error; 3D-OCT = 3-dimensional optical coherence tomography.

## Discussion

The new combinations generated using parameters provided by Catalys 3D-OCT enhanced the predictive accuracy comparing with the existing IOL formulas. This is the first study to investigate LMP provided by Catalys 3D-OCT as a new variable to predict the effective lens position (ELP) and develop a new IOL formula using LMP and the posterior part of cataractous lens.

The 3D-OCT used in our study obtained images with a wavelength of 830-nm and provided axial and lateral resolutions of 30 and 15 μm, respectively. This technique has the advantage of showing each structure covered by a liquid optic interface with a clear aperture of 13.5 mm. OCT scans from the corneal epithelium to the posterior capsule of the lens can be viewed on one image because of a greater depth of field. This machine could restructure the lens by detecting the exposed anterior and posterior surface of the lens; therefore, the surgeon can notice the position of the lens equator on the sagittal or axial view of the lens. Using estimated lens equator points, the manufacturer developed a new parameter, the line connecting equator points, and the distance from the anterior surface of the cornea to the equator line was defined as the LMP.

We investigated five parameters provided by 3D-OCT (ACD, aLT, pLT, LMP, and ASL) and developed six new equations for ELP. The combination of AL, LMP, and pLT performed best for ELP prediction and also showed the highest predictive accuracy as an IOL power calculation formula. The Haigis formula was the best open-source formula, even when using a study-specific single optimization of the Haigis A-constant [11]. By contrast, the prediction error of the Haigis formula was changed by preoperative LT, and this study revealed a similar result and indicates that a more myopic result is obtained when the preoperative cataractous lens becomes thicker. Considering ACD alone without consideration of LT, it is possible to underestimate the size of the anterior segment if the cataract progresses and cataractous lens becomes too thick. The new formulas that use LMP, rather than ACD or LT as a variable to ACD have the advantage of not being affected by LT.

Almost all theoretical formulas for IOL power calculation are based on the use of a simplified eye model with a thin cornea and IOL model [2]. According to such an approach, the power of the IOL can be easily calculated using the Gauss equation in paraxial optics [12]. The ELP is back-calculated as the effective ACD for “predicting” the actual postoperative refraction of a given data set. Therefore, the ELP is formula-dependent and does not need to reflect the real postoperative IOL position in the anatomic sense [13]. Models based on statistically analyzed relationships between some or all of the previously mentioned preoperative measurements of the eye and postoperative IOL position have been used to predict the ELP in preoperative settings. In 1975, Fyodorov et al. [10] derived an equation based on an individual eye’s Keratometry and axial length to estimate the ELP. Third-generation formulas, including the Hoffer Q [12], Holladay 1 [13], and SRK/T formulas [14], use AL and corneal power to predict ELP and IOL power calculation. The ACD could be measured accurately after the development of slit-scan technology and a fourth-generation formula was developed. The Haigis formula uses AL and ACD values to estimate the ELP [15].

Recent developments in anterior segment-optical coherence tomography have made it possible to measure the lens thickness, and even to subdivide the lens thickness structure. The Olsen formula includes the concept of the C constant [16], which is related to the IOL type that is determined as the mean value calculated from representative samples. Goto et al. [17] introduced the angle-to-angle depth and concluded that three preoperative variables (the angle-to-angle depth, ACD, and AL) predicted the postoperative IOL position better than the Haigis formula. Hirnschall et al. [18] measured the intraoperative anterior capsule after capsular tensing ring implantation following phacoemulsification and developed a new regression equation (intraoperative anterior lens capsule, ACD, and AL) to predict the postoperative IOL position. Satou et al. [19] concluded that the equatorial surface depth and the posterior surface depth provided by a swept-source OCT with a wavelength of 1310-nm were highly correlated with postoperative IOL position. A new formula proposed by Shammas considers the distance from the corneal apex to the anterior surface of the endonucleus and the thickness of the endonucleus measured using optical low-coherence reflectometry [2]. These authors concluded that newer formulas show a better predictability than prior formulas, including the Haigis formula.

Femtosecond laser-assisted cataract surgery enhanced the precision and reproducibility of clear corneal incision and capsulotomy. The total amount of ultrasound energy is also reduced compared to conventional cataract surgery [20]. However, in a comparison of predictive accuracy, which is the most important assessment, recent large-scale studies have shown worse results compared to normal cataract surgery [21, 22]. Manning et al. [21] found that the mean absolute error was 0.43 diopters for femtosecond laser-assisted cataract surgery and 0.40 diopters for the manual group. Another study also found that the mean absolute error was 0.41 diopters for femtosecond laser-assisted cataract surgery and 0.35 diopters for conventional cataract surgery [22]. Femtosecond laser-assisted cataract surgery revealed lower percentages of eyes within ± 0.5 diopters of the mean error in both studies. Based on the above results, Ewe et al. [22] concluded that femtosecond laser-assisted cataract surgery is less cost-effective. The new IOL formula developed in this study improves the predictive accuracy and will contribute to the emergence of femtosecond laser-assisted cataract surgery.

Our study has some limitations. Only one type of IOL was implanted. As ELP or the postoperative IOL position is influenced by IOL material or IOL design, different types of IOL should be evaluated. Secondly, the sample size of 145 eyes was relatively small. In order to develop reliable equations to predict the ELP, it would be necessary to enroll larger number of patients, especially with short and long AL. Finally, we obtained a value of the axial length and corneal radius from PCI. Recently, swept-source optical coherence tomography (SS-OCT) technique was introduced to measure axial length. SS-OCT with 1050–1060 nm light has also been used to obtain biometric measurements because a higher source light penetration was achieved than with PCI at 780-nm light [23]. They also use a different measurement technique for the corneal radius. Future studies will need to find an ideal combination with Catalys 3D-OCT or analyze the predictive accuracy when using the axial length and corneal radius measured on a new biometer.

In conclusion, the combination of AL, LMP, and posterior LT showed the best predictability for both ELP and refractive outcomes. Our new IOL formula using LMP and posterior LT provided by Catalys 3D-OCT as variables will help to enhance the predictive accuracy of femtosecond laser-assisted cataract surgery.

## References

[1] Lundstrom M, Pesudovs K. Questionnaires for measuring cataract surgery outcomes. J Cataract Refract Surg, 2011;37: 945–959. 10.1016/j.jcrs.2011.03.010 21511158

[2] Shammas HJ, Shammas MC. Improving the preoperative prediction of the anterior pseudophakic distance for intraocular lens power calculation. J Cataract Refract Surg. 2015;41: 2379–2386. 10.1016/j.jcrs.2015.05.032 26703486

[3] Lansingh VC, Carter MJ, Martens M. Global cost-effectiveness of cataract surgery. Ophthalmology. 2007;114: 1670–1678. 10.1016/j.ophtha.2006.12.013 17383730

[4] Nguyen P, Chopra V. Applications of optical coherence tomography in cataract surgery. Curr Opin Ophthalmol. 2013;24: 47–52. 10.1097/ICU.0b013e32835aee7b 23197267

[5] Kohnen T, Thomala MC, Cichocki M, Strenger A. Internal anterior chamber diameter using optical coherence tomography compared with white-to-white distances using automated measurements. J Cataract Refract Surg. 2006;32: 1809–1813. 10.1016/j.jcrs.2006.08.023 17081862

[6] Pinero DP, Plaza Puche AB, Alio JL. Corneal diameter measurements by corneal topography and angle-to-angle measurements by optical coherence tomography: evaluation of equivalence. J Cataract Refract Surg. 2008;34: 126–131. 10.1016/j.jcrs.2007.10.010 18165092

[7] Pinero DP, Plaza AB, Alio JL. Anterior segment biometry with 2 imaging technologies: very-high-frequency ultrasound scanning versus optical coherence tomography. J Cataract Refract Surg. 2008;34: 95–102. 10.1016/j.jcrs.2007.08.033 18165088

[8] Shen P, Ding X, Congdon NG, Zheng Y, He M. Comparison of anterior ocular biometry between optical low-coherence reflectometry and anterior segment optical coherence tomography in an adult Chinese population. J Cataract Refract Surg. 2012;38: 966–970. 10.1016/j.jcrs.2011.12.031 22624895

[9] Colenbrander MC. Calculation of the power of an iris clip lens for distant vision. Br J Ophthalmol. 1973;57: 735–740. 10.1136/bjo.57.10.735 4784206 PMC1215153

[10] Fyodorov SN, Galin MA, Linksz A. Calculation of the optical power of intraocular lenses. Invest Ophthalmol Vis Sci. 1975;14: 625–628. 1150402

[11] Melles RB, Holladay JT, Chang WJ. Accuracy of intraocular lens calculation formulas. Ophthalmology. 2017;125: 169–178. 10.1016/j.ophtha.2017.08.027 28951074

[12] Hoffer KJ. The Hoffer Q formula: a comparison of theoretic and regression formulas. (errata, 20, 677 (1994)) J Cataract Refract Surg. 1993;19: 700–712. 10.1016/s0886-3350(13)80338-0 8271165

[13] Holladay JT, Prager TC, Chandler TY, Musgrove KH, Lewis JW, Ruiz RS. A three-part system for refining intraocular lens power calculations. J Cataract Refract Surg. 1988;14: 17–24. 10.1016/s0886-3350(88)80059-2 3339543

[14] Retzlaff JA, Sanders DR, Kraff MC. Development of the SRK/T intraocular lens implant power calculation formula. (erratum, 528) J Cataract Refract Surg. 1990;16: 333–340. 10.1016/s0886-3350(13)80705-5 2355321

[15] Haigis W. Occurrence of erroneous anterior chamber depth in the SRK/T formula. J Cataract Refract Surg. 1993;19: 442–446. 10.1016/s0886-3350(13)80325-2 8501649

[16] Olsen T, Hoffmann P. C constant: new concept for ray tracing-assisted intraocular lens power calculation. J Cataract Refract Surg. 2014;40: 764–773. 10.1016/j.jcrs.2013.10.037 24767910

[17] Goto S, Maeda N, Koh S, Ohnuma K, Hayashi K, Iehisa I, et al Prediction of postoperative intraocular lens position with angle-to-angle depth using anterior segment optical coherence tomography. Ophthalmology. 2016;123: 2474–2480. 10.1016/j.ophtha.2016.09.005 27769585

[18] Hirnschall N, Amir-Asgari S, Maedel S, Findl O. Predicting the postoperative intraocular lens position using continuous intraoperative optical coherence tomography measurements. Invest Ophthalmol Vis Sci. 2013;54: 5196–5203. 10.1167/iovs.13-11991 23761092

[19] Satou T, Shimizu K, Tsunehiro S, Igarashi A, Kato S, Koshimizu M, et al Relationship between crystalline lens thickness and shape and the identification of anterior ocular segment parameters for predictiong the intraocular lens position after cataract surgery. Biomed Res Int. 2019: 3458548 10.1155/2019/3458548 31360711 PMC6644274

[20] Dick HB, Schultz T. A review of laser-assisted versus traditional phacoemulsification cataract surgery. Ophthalmol Ther. 2017;6: 7–18. 10.1007/s40123-017-0080-z 28188490 PMC5449299

[21] Manning S, Barry P, Henry Y, Rosen P, Stenevi U, Young D, et al Femtosecond laser-assisted cataract surgery versus standard phacoemulsification cataract surgery: Study from the European Registry of Quality Outcomes for Cataract and Refractive Surgery. J Cataract Refract Surg. 2016;42: 1779–1790. 10.1016/j.jcrs.2016.10.013 28007110

[22] Ewe SY, Abell RG, Oakley CL, Lim CH, Allen PL, McPherson ZE, et al A comparative cohort study of visual outcomes in femtosecond laser-assisted versus phacoemulsification cataract surgery. Ophthalmology. 2016;123: 178–182. 10.1016/j.ophtha.2015.09.026 26526634

[23] Shammas HJ, Ortiz S, Shammas MC, Kim SH, Chong C. Biometry measurements using a new large-coherence-length swept-source optical coherence tomographer. J Cataract Refract Surg. 2016;42: 50–61. 10.1016/j.jcrs.2015.07.042 26948778

